# SRComp: Short Read Sequence Compression Using Burstsort and Elias Omega Coding

**DOI:** 10.1371/journal.pone.0081414

**Published:** 2013-12-13

**Authors:** Jeremy John Selva, Xin Chen

**Affiliations:** Division of Mathematical Sciences, School of Physical and Mathematical Sciences, Nanyang Technological University, Singapore, Singapore; Kansas State University, United States of America

## Abstract

Next-generation sequencing (NGS) technologies permit the rapid production of vast amounts of data at low cost. Economical data storage and transmission hence becomes an increasingly important challenge for NGS experiments. In this paper, we introduce a new non-reference based read sequence compression tool called SRComp. It works by first employing a fast string-sorting algorithm called burstsort to sort read sequences in lexicographical order and then Elias omega-based integer coding to encode the sorted read sequences. SRComp has been benchmarked on four large NGS datasets, where experimental results show that it can run 5–35 times faster than current state-of-the-art read sequence compression tools such as BEETL and SCALCE, while retaining comparable compression efficiency for large collections of short read sequences. SRComp is a read sequence compression tool that is particularly valuable in certain applications where compression time is of major concern.

## Introduction

Next-generation sequencing (NGS) technologies are gradually replacing Sanger sequencing as the dominant sequencing technologies and are yielding a revolutionary impact on genetics and biomedical research. These technologies can rapidly sequence DNA on the gigabase scale in a single run, thus generating hundreds or even thousands of gigabases in just a few days. Management, storage and analysis of such large amounts of sequencing data, however, pose many daunting bioinformatics challenges. For example, as NGS data output continues to grow exponentially, there is a pressing need for a fast and efficient approach to compressing NGS read sequences for economical data storage and transmission [1].

A number of tools have been developed to compress NGS data in recent years [2]–[10]. Most of them are aimed at compression of data files in the FASTQ format, which is the most-used data format for NGS data. Although read DNA sequences are mixed with their associated quality scores in FASTQ files, they are usually processed separately and compressed using different approaches. For example, a reference-based approach is often used to compress read DNA sequences. This approach first aligns reads to a known reference genome sequence and then encodes reads compactly as genomic positions and any aligning differences [5], [7], [8], [11], [12]. While highly efficient, it is inapplicable to datasets for which no appropriate reference genome sequences are available (e.g., in metagenomic or *de novo* sequencing). Furthermore, the reference-based compressed data are at high risk of being inaccessible once the reference genome sequence used for compression is lost [13].

There also exist many methods to compress read sequences without the help of a reference genome [3], [4], [6], [10], [13]. Interestingly, most of the efficient methods adopt a common compression strategy; that is, the input reads are first reorganized to maximize locality of redundancy and then passed to a general-purpose compressor such as gzip (J. Gailly and M. Adler, http://www.gzip.org) or bzip2 (J. Seward, http://www.bzip.org). A variety of specific techniques have been explored to reorganize reads. Wan and Asai first showed that sorting reads in lexicographical order can improve compression efficiency [10]. ReCoil aimed to group together read sequences that share many 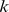-mers [13]. BEETL instead permuted the read sequences by using the Burrows-Wheeler transform (BWT) [3]. In a recent method, SCALCE tried to group together the reads that share common string ‘signatures’, i.e., the core substrings that are derived from the locally consistent parsing (LCP) [6]. All the above methods assume that the order of reads is not important, and so there is no need to preserve it.

In this paper, we introduce a new approach for non-reference based compression of read DNA sequences. It works in a similar way to the method proposed in [10], in which reads are first sorted in lexicographical order and then encoded. In order to further increase compression speed and efficiency, we make improvements in the following two ways. First, we use a fast string-sorting algorithm called burstsort [14] to sort read sequences. In contrast, radix sort and quicksort were used in [10]. Second, we apply Elias omega-based integer coding [15] to the sorted read sequences rather than using the general-purpose compressors gzip or bzip2 as in [10]. Our approach is implemented in a software tool called SRComp, and has been benchmarked on four NGS datasets of sizes up to 7 GB. The experimental results show that SRComp can run 5–35 times faster than current state-of-the-art read sequence compression tools, such as BEETL and SCALCE, while retaining comparable compression efficiency.

## Methods

In this section, we introduce the algorithms burstsort and Elias omega-based integer coding in detail. They constitute the two major components of our new read sequence compression approach.

### Sorting reads

Before sorting reads, we first convert every occurrence of the ambiguous base N in the read sequences into the (randomly selected) base G. Consequently, the read sequences to be sorted below are made of only four bases {A, C, G, T}.

As every read to be sorted is represented as a string over an alphabet of four letters {A, C, G, T}, the first aim in this study is to find a fast string-sorting algorithm that can sort a large collection of reads in lexicographic order. Many sorting algorithms have been proposed in the literature such as insertion sort, mergesort and quicksort. Although these algorithms can be directly applied to sort strings, their computing efficiency and performance vary significantly. In the previous study of [10], radix sort and quicksort were used to sort reads and the experimental results showed that sorting time made up a substantial proportion of the total read compression time. Thus, a faster string-sorting algorithm is highly desired to speed up the whole read compression process, especially when hundreds of millions of reads are handled.

**Figure 1:**
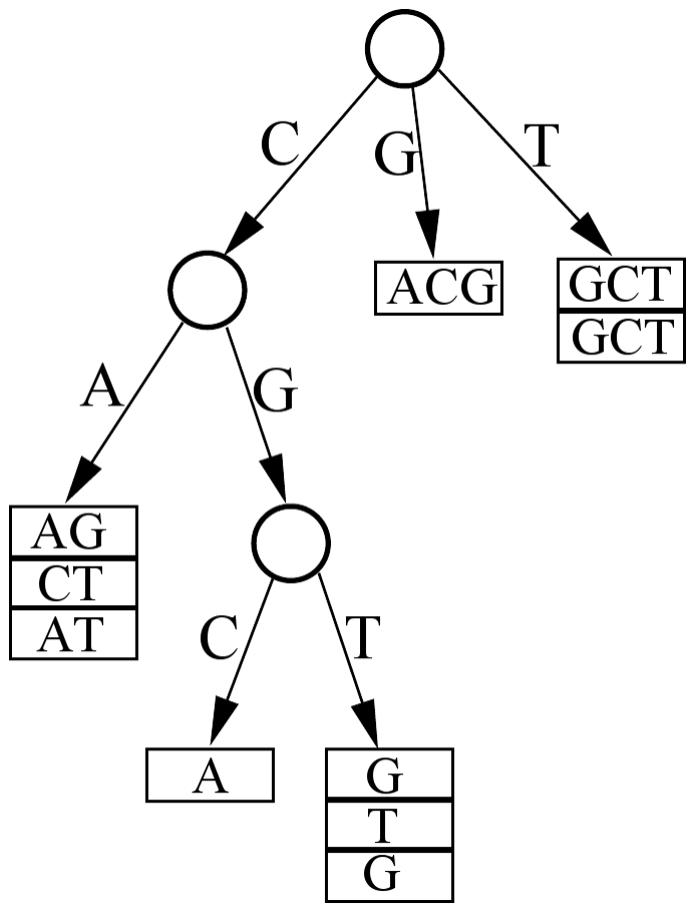
A burst trie built from ten read sequences. The ten read sequences used are {CGCA, CAAG, TGCT, CGTG, CGTT, GACG, CACT, TGCT, CAAT, CGTG}. This burst trie has three trie nodes and five buckets. The maximum capacity of a bucket is assumed to be three read sequences.

For fast sorting of read sequences, we choose a cache-conscious string-sorting algorithm called burstsort. Burstsort was introduced by Sinha and Zobel in 2004, and has been demonstrated to be around twice as fast as the best of the other string-sorting algorithms [14]. To sort a collection of read sequences, burstsort starts with inserting each read sequence into a burst trie – a variant of trie in which sufficiently small subtrees are represented as buckets (see Figure 1). Specifically, read sequences are retrieved from the main memory in a sequential manner. Once a read sequence is retrieved, it is inspected one base after another until it can be assigned to a bucket. Buckets that have reached the maximum capacity are then burst into new subtries, leading to more buckets created at depth of one level deeper. To output read sequences in lexicographical order, the read sequences within each bucket are first sorted using a method that is efficient for sorting small datasets (e.g., insertion sort or quicksort), and then the constructed burst trie is traversed depth-first and from left to right in each trie node. As all the read sequences within a bucket share the same prefix, their comparisons during sorting can simply start from the base position 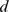 if the bucket is at depth 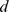.

Burstsort is a cache-oblivious string-sorting algorithm, as it takes advantage of the cache system of a CPU (i.e., the local memory of frequently accessed data) to avoid expensive memory access operations and thus to improve efficiency. It is faster at sorting primarily due to a very low rate of cache misses. First, burstsort accesses read sequences serially and inserts them into buckets one after another, which means that a read sequence is fully processed before proceeding to the next. By contrast, radix sort proceeds by inspecting the first base of every read sequence before the second base of any read sequence, so that each read sequence needs to be re-fetched from main memory for each base. Thus, it can be seen that burstsort allows memory access much more localized than radix sort, leading to significantly fewer cache misses. Second, burstsort bursts a bucket into new subtries whenever it is full, so every bucket is maintained at a small size. As a result, the read sequences within each bucket can be fully sorted inside fast cache memory, which do not incur any unnecessary L2 cache misses.

There exist two variants of burstsort, one using linked lists to represent buckets and the other using dynamic arrays [14]. Implementations of these two variants can be found at http://www.cs.mu.oz.au/rsinha/resources/source/sort/allsorts/allsorts.zip.

### Sorting large collections of reads

We have assumed until now that the whole collection of reads can be resident in main memory for sorting and encoding. However, today's NGS data output, even from a single experiment, often far exceeds the amount of main memory available in our computing environment. Thus, a critical issue is how to sort a large collection of read sequences that cannot fully reside in main memory.

To solve this issue, we choose a simple approach which can be divided into two phases. In the first phase, we create a number of temporary files on the local hard disk and allocate each input read into one of the temporary files based on its first few bases (in a way similar to the most significant digit (MSD) radix sort). The number of temporary files to be created is carefully determined to ensure that every temporary file is small enough to fit in main memory for read sorting. During the second phase, read sequences in each temporary file are fetched to main memory and sorted with the burstsort algorithm as described above. The sorted read sequences are then encoded (see the next subsection) and the encoded bitstreams are written to the final compressed file before proceeding to the next temporary file. As these temporary files are sorted and encoded independently, the above two-phase approach additionally allows for easy implementations of parallel compression and decompression.

### Encoding a collection of sorted reads

After the sorting step presented above, we now have a collection of read sequences in lexicographical order. Our second aim in this study is to find a fast and efficient algorithm to encode these sorted read sequences. In the previous study of [10], the general-purpose compression tools gzip and bzip2 were tested. These tools, however, were not originally developed to capture the intrinsic sequence pattern of a collection of sorted reads. Thus, a significant improvement in compression efficiency could be achieved with a domain-specific encoding algorithm.

**Figure 2:**
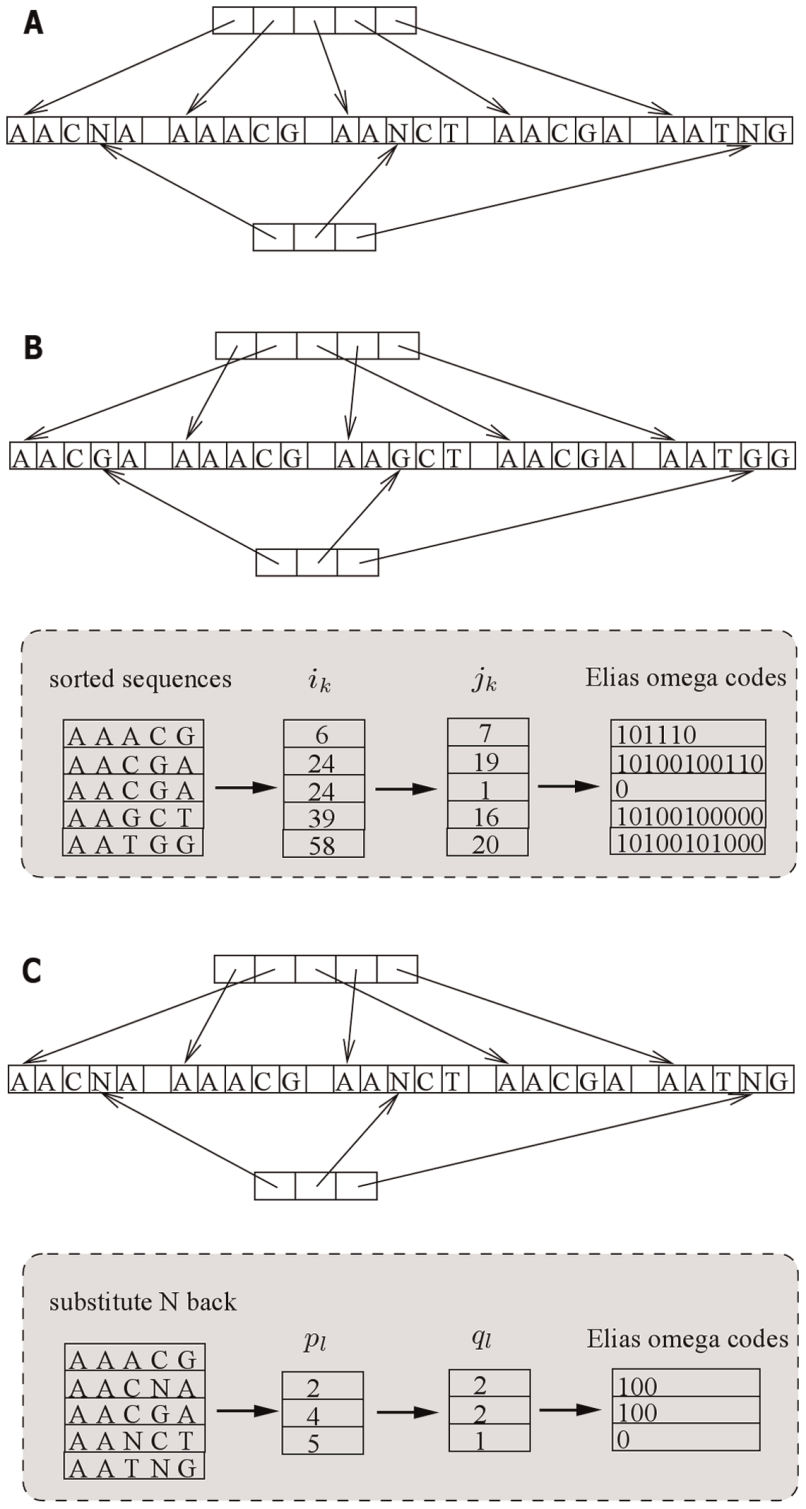
The algorithm overview for compression. (A) After five input read sequences are loaded in memory, we build two arrays of pointers. The first array (upper) contains pointers each of which points to a read sequence, whereas the second array (lower) contains pointers each of which points to an occurrence of the ambiguous base N. (B) Before burstsort starts, all the ambiguous bases are substituted with base G. During sorting, read sequences remain at the same physical place in memory and only their respective pointers in the first array are moved into sort order. At the end, read sequences are retrieved in order via the first pointer array. (C) Once the encoding of ordered read sequences is completed, all the ambiguous bases are substituted back via the second pointer array, which enables finding the location of every ambiguous base within the collection of sorted read sequences.

To this end, we propose a read-encoding algorithm based on a method of encoding a monotone sequence of integers (see Figure 2B). Firstly, each read sequence is converted into an integer by a trivial two-bit coding of four bases: A
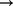
00, C
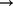
01, G
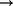
10, and T
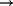
11. Consequently, a collection of 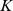 reads will give rise to a sequence of integers 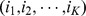. As the input reads are sorted in lexicographical order, these integers are not decreasing; that is, 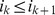 holds true for 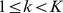. Meanwhile, it is worth noting that every read sequence can be unambiguously recovered from its integer representation provided that the read length is known.

Next, we convert the above non-decreasing sequence of integers into another sequence of integers 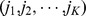, where 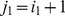 and 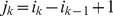 for 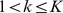. That is, each 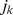 is the difference between two consecutive elements of 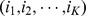 plus one. Since 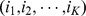 is a non-decreasing sequence with the first element 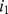 being a non-negative integer, every 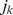 shall take on a positive integer. Note that these two sequences of integers are equivalent in the sense that one sequence can be unambiguously recovered from the other. We added one to every difference in the above just because there is no Elias omega code for the integer zero (see the next paragraph for Elias omega coding).

In the last step, we encode the sequence of positive integers 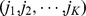 by using Elias omega coding. Elias omega coding is a universal code developed by Peter Elias [15], which represents a positive integer by a variable-length self-delimiting bitstream. Encoding a positive integer 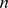 is done recursively in the following steps:

1. Initialize the bitstream to a single bit 0.
2. If 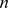 equals 1, stop; otherwise, prefix the bitstream with the least significant 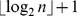 bits of the binary representation of 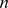.
3. Let 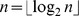, and then go to step 2 again.

For example, if we encode the integer 17 with Elias omega coding, the resulting bitstream is 10100100010, using a total of 11 bits. To decode an Elias omega-coded bitstream back to the integer 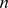, we may use the following steps:

1. Initialize 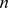 to 1.
2. Read the next bit from the given Elias omega-coded bitstream. If it is 0, stop; otherwise, read 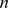 more bits.
3. Let 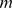 be the integer such that the least significant 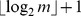 bits of the binary representation of 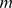 are the same as the 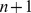 bits just read in step 2.
4. Let 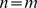, and then go to step 2 again.

For example, if the input Elias omega-coded bitstream is 10100100010, then 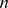 will take on three different values of 1, 2 and 17 successively during the execution of the above steps. At the end, the decoded integer is 17.

In general, Elias omega coding requires only a few bits to encode small integers. As the integer value increases, more and more bits would be needed. Indeed, the distribution of integers in the sequence 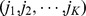 tends to be skewed towards smaller integers, being particularly true when compared to the sequence 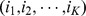, as we show in Figure S1. Therefore, better data compression is expected when we choose to encode the sequence 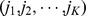 instead of the sequence 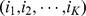.

### Encoding ambiguous bases

The primary aim of this study is to develop a lossless compression approach for a large collection of short read sequences. Hence, it is also necessary to encode all the occurrences of the ambiguous base N of read sequences into the final compressed file.

Recall that we substituted the base G for every ambiguous base N before the sorting of reads. In order to undo these substitutions at the decompression stage, we keep track of all the ambiguous bases and record where their substituted base Gs are within the collection of sorted reads (see Figure 2C). Then, we obtain a sequence of integers 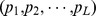, where 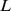 is the total number of ambiguous bases and each 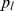 (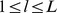) indicates that the 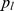-th base G within the collection of sorted reads originally comes from an ambiguous base N. It is obvious that 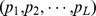 is an increasing sequence of positive integers; that is, we have 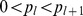 for 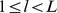.

We encode the sequence of integers 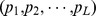 in a similar way to the sequence 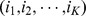. First, 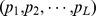 is converted into another sequence of integers 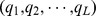 such that 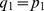 and 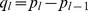 for 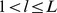. Then, we encode 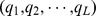 by using Elias omega coding. At the decompression stage, after the collection of *sorted* reads and the sequence of integers 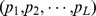 are decoded, the 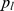-th base G within the collection of sorted reads is finally substituted back by an ambiguous base N.

## Results

We implemented the read sequence compression approach described above in a software tool called SRComp, which is freely available at http://www1.spms.ntu.edu.sg/~chenxin/SRComp. For the implementation of burstsort, we have made use of the source code from http://www.cs.mu.oz.au/~rsinha/resources/source/sort/allsorts/allsorts.zip. The bucket capacity used is 8192 pointers of strings.

### Datasets

To evaluate the performance of SRComp in compressing short read sequences, we carried out comparative experiments on four different datasets downloaded from the DDBJ Sequence Read Archive (SRA). Their respective accession IDs are SRR014437, SRX001540, SRX006998 and SRX011353 (see Table S1). Among these four datasets, the first two datasets, as well as one run from the fourth dataset (i.e., SRR027520), were already employed in several previous studies to test a variety of read sequence compression tools [2], [3], [10], [13]. Table 1 gives some basic statistics for these datasets. One might notice that read length is fixed for each dataset. This is because SRComp, as well as BEETL and SCALCE, is not applicable to reads of variable length.

**Table 1:** Some basic statistics for datasets used in the experiments.

accession ID	species	read length	read count	ambiguous base count	file size
SRR014437	S. cerevisiae	28	11,759,238	2,875,210	341,017,902
SRX001540	H. sapiens	36	192,132,426	13,697,303	7,108,899,762
SRX006998	A. melanoleuca	45	162,714,366	764,334	7,484,860,836
SRX011353	H. sapiens	76	91,299,128	20,532,746	7,030,032,856

As the paired-end reads from the experiment SRX006998 are of different length, we include in this dataset only reads from one end.

All experiments were conducted on a cluster of Intel Xeon X5355 machines with 2.66 GHz CPU and 10 GB RAM. As SRComp currently does not support multi-threading, it can be run only using a single thread. In contrast, SCALCE can make use of multi-cores and multi-threads. Therefore, to measure each program's running time we used the Linux command time to report the amount of CPU time consumed (i.e., the user time plus the sys time) instead of the elapsed wall clock time. Times are averaged over five runs for each test.

### Sorting large collections of reads

In the first experiment, we demonstrate how fast the burstsort algorithm can sort a large collection of short reads and also compare its performance with other well-known string-sorting algorithms. In total, seven algorithms are compared, including three variants of radix sort, two variants of quicksort and two variants of burstsort. Details of these algorithms can be found in [14]. The two variants of burstsort differ only by their data structures used to represent buckets (i.e., one using linked lists and the other using dynamic arrays).

Table 2 shows the sorting times for the above seven algorithms. We can see that burstsort using dynamic arrays is the fastest for all of the datasets tested. It is typically more than twice as fast as MSD radix sort and the conventional quicksort. Multikey quicksort was the second fastest, but by a significant margin (22–31%) is still slower than burstsort using dynamic arrays. Based on these observations, we choose burstsort using dynamic arrays as the read sorting algorithm implemented in our software SRComp.

**Table 2:** Comparison of CPU time needed to sort large collections of reads.

	radix sort			quicksort		burstsort	
accession ID	MBM	MSD	Adaptive	Quicksort	Multikey	List	Array
SRR014437	14.0s	15.3s	12.0s	17.7s	8.2s	8.8s	6.4s
SRX001540	5m20s	5m23s	4m06s	6m54s	3m03s	3m05s	2m19s
SRX006998	4m42s	4m38s	3m36s	6m00s	2m46s	2m46s	2m09s
SRX011353	3m01s	2m52s	2m20s	3m38s	1m55s	1m53s	1m34s

Designations of these algorithm variants can be found in [14]. Times are averaged over five runs.

### Encoding large collections of sorted reads

In the second experiment, we test the efficiency of Elias omega-based integer coding to encode a collection of read sequences which are already sorted in lexicographical order. In particular, we investigate whether Elias omega coding could achieve better compression than gzip and bzip2, since the latter two were previously employed in [10] for the same task.

Our experimental results are summarized in Table 3, where the compression rate is quantified as bits encoded per base (bpb). As we can see, both gzip and bzip2 can obtain better compression than the trivial 2-bit encoding method, which is in agreement with the previous observations in [10]. In comparison, Elias omega coding can still make significant improvements, achieving substantial reduction of compression bit rates by as much as 12–31%. Moreover, Elias omega coding can run around four times faster than gzip and bzip2. These results indicate that Elias omega coding is a fast and efficient approach to encoding a large collection of sorted read sequences.

**Table 3:** Comparison of compression CPU time and bit rates of Elias omega coding to gzip and bzip2 on sorted read sequences.

	gzip		bzip2		Elias omega coding	
accession ID	c-time	bpb	c-time	bpb	c-time	bpb
SRR014437	49.3s	1.50	3m28s	1.63	14.2s	1.12
SRX001540	18m47s	1.61	24m20s	1.72	5m14s	1.21
SRX006998	24m10s	1.87	25m20s	1.86	6m00s	1.45
SRX011353	24m21s	1.99	19m02s	1.91	5m57s	1.68

In the above, c-time means compression CPU time and bpb denotes bits per base. Times are averaged over five runs.

### Comparison to previous read sequence compression tools

In this last experiment, we evaluate the overall performance of SRComp in compression efficiency and speed. Besides the two general-purpose compressors, gzip and bzip2, we also carry out tests on two recently developed state-of-the-art read sequence compression tools, BEETL [3] and SCALCE [6], for a comprehensive comparative study.

Like SRComp, both BEETL and SCALCE adopt a two-stage procedure for read sequence compression. In brief, BEETL first computes the Burrows-Wheeler Transform (BWT) for a collection of read sequences and then passes the resulting permuted sequences to a standard text compressor such as gzip or PPM (Prediction by Partial Matching). PPM is an adaptive statistical data compression technique based on context modeling and prediction [16]. In our experiments below, a variant called PPMd is actually used with BEETL, because it is more efficient than gzip for compressing BWT-permuted sequences as demonstrated in [3].

In comparison, SCALCE first groups read sequences that share the same ‘core’ substrings and then passes the reads in each group to a standard text compressor. The core substrings are identified by using a combinatorial pattern matching technique called *locally consistent parsing* (LCP), which aims to identify the ‘building blocks’ of strings. SCALCE is designed primarily for compressing FASTQ files, which include not only read sequences, but also read names and quality scores. With a proper option setting, however, it can be tuned to compress read sequences alone. In addition, SCALCE uses gzip as the default text compressor, which is also used in our experiments below.

Table 4 shows the running times and compression bit rates achieved by a variety of read sequence compression tools. A remarkable result that can be clearly seen is that SRComp can run very fast. For compression, it is around twice as fast as the best of the other tools tested, 5–8 times faster than SCALCE and 19–35 times faster than BEETL. Specifically, it takes SRComp only about 8 minutes to compress a dataset of 7 gigabases while SCALCE and BEETL need up to 50 minutes and 4 hours, respectively. It is not surprising that BEETL is the slowest among these tools because it relies on a computationally demanding transform (i.e., Burrows-Wheeler transform) and moreover, it works on external memory with frequent access to files held on disk. To compare decompression time, SRComp is inferior only to gzip, but still about 3–4 times faster than SCALCE. In terms of compression efficiency, SRComp performs the best for the first two datasets, but worse than BEETL and SCALCE by a relatively small margin for the third and forth datasets. As expected, the compression bit rates of gzip and bzip2 are all far above 2 bpb, indicating that the general-purpose text compressors are not suited for compressing read sequences. BEETL appears more efficient than SCALCE for all the datasets tested here, which is, however, opposite to a previous observation in [6]. One reason for this discrepancy might be that BEETL was tested in combination with bzip2 in [6] instead of PPMd which we used here. These results show that SRComp can retain state-of-the-art compression efficiency using substantially less CPU computing time.

**Table 4:** Comparison of compression performance of SRComp to gzip, bzip2, BEETL and SCALCE.

accession ID	software	c-time	bpb	d-time	p-mem
SRR014437	gzip	1m36s	2.56	3.4s	2.6
	bzip2	52.0s	2.31	19.0s	28.4
	BEETL	6m39s	1.18	47m29	36.8
	SCALCE	2m47s	1.25	46.2s	7604
	SRComp	20.6s	1.12	9.5s	2310
SRX001540	gzip	32m15s	2.51	1m18s	2.6
	bzip2	18m21s	2.31	6m45s	28.5
	BEETL	2h49m10s	1.25	12h54m5s	35.3
	SCALCE	51m50s	1.26	15m0s	21915
	SRComp	7m33s	1.21	3m43s	6992
SRX006998	gzip	34m31s	2.47	1m17s	2.6
	bzip2	18m55s	2.28	6m57s	28.5
	BEETL	3h26m48s	1.38	16h40m27s	33.4
	SCALCE	49m04s	1.40	15m25s	21842
	SRComp	8m09s	1.45	4m32s	6842
SRX011353	gzip	30m33s	2.44	1m18s	2.6
	bzip2	17m52s	2.24	6m39s	28.5
	BEETL	4h22m47s	1.45	12h13m34s	40.0
	SCALCE	37m01s	1.47	13m55s	21767
	SRComp	7m31s	1.68	4m56s	7244

BEETL is run in combination with PPMd, and SCALCE in combination with gzip. In the above, p-mem and d-time denote the compression peak memory usage (megabytes) and decompression CPU time, respectively. Times are averaged over five runs.

### Evaluation on various simulated datasets

To extensively test our approach, we generated six simulated datasets of varying read lengths and varying coverage depths for the NA18507 human genome (see Table 5). The initial read dataset is from the experiment SRX016231, which contains a total of 1.4 billion 100 bp reads from 37 runs corresponding to 44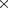 genome coverage. To obtain the first simulated dataset, we took the first 35 bases of every read in the initial read dataset to form a new read sequence, thereby resulting in 1.4 billion 35 bp reads and 15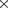 genome coverage. For the second simulated dataset, we used not only the first 35 bases but also the next 35 bases of the initial 100 bp reads to form reads of length 35 bp, resulting in 2.8 billion 35 bp reads and 31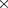 genome coverage. Similarly, we generated the other four datasets for reads of length 50 bp, 75 bp and 100 bp, respectively.

**Table 5:** Evaluation of SRComp on simulated datasets of varying read lengths and genome coverage depths.

dataset	software	c-time	bpb	d-time	p-mem
35 bp reads	gzip	3h50m9s	2.53	10m14s	2.6
(15 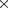 coverage)	bzip2	2h10m31s	2.32	49m38s	28.4
	SCALCE	6h11m3s	1.14	1h48m41s	22013
	SRComp	54m47s	1.06	23m21s	19432
35 bp reads	gzip	7h37m34s	2.53	19m51s	2.6
(31 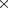 coverage)	bzip2	4h18m49s	2.33	1h36m22s	32.0
	SCALCE	12h8m55s	1.10	–	22047
	SRComp	1h47m22s	0.97	44m18s	25939
50 bp reads	gzip	5h17m14s	2.47	13m26s	2.6
(22 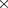 coverage)	bzip2	3h3m54s	2.27	1h9m28s	28.4
	SCALCE	7h16m15s	1.05	2h28m8s	21883
	SRComp	1h12m41s	1.25	38m22s	17708
50 bp reads	gzip	10h34m15s	2.48	29m16s	2.6
(44 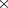 coverage)	bzip2	6h4m46s	2.29	2h17m11s	32.0
	SCALCE	14h17m4s	1.07	–	21977
	SRComp	2h23m35s	1.19	1h16m53s	30011
75 bp reads	gzip	7h46m1s	2.43	19m59s	2.6
(33 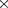 coverage)	bzip2	4h31m47s	2.23	1h44m40s	28.5
	SCALCE	9h20m1s	0.97	3h32m22s	21893
	SRComp	1h45m18s	1.41	1h4m8s	16762
100 bp reads	gzip	10h18m54s	2.40	26m45s	2.6
(44 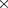 coverage)	bzip2	5h57m42s	2.21	2h12m40s	28.5
	SCALCE	11h26m48s	0.97	4h40m40s	21905
	SRComp	2h20m31s	1.52	1m29m37s	21503

SCALCE's decompression crashed on two datasets tested, one consisting of 35 bp reads at 31 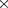 coverage and the other consisting of 50 bp reads at 44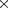 coverage. Hence, their corresponding decompression times are indicated by a hyphen mark (−) in the above table. Times are averaged over five runs.

We ran gzip, bzip2, SCALCE and SRComp on these simulated datasets, and summarized the experimental results in Table 5. BEETL is excluded from our tests here because it is expected to run extremely slow for such large datasets of size up to 140 gigabases. From Table 5, we can see similar performance of these tools to our previous experiments. First, SRComp can compress consistently more efficiently than gzip and bzip2. Compared to SCALCE, SRComp is more efficient for short reads but less efficient for long reads. Second, SRComp can run very fast in both compression and decompression. It is around 5–6 times faster than SCALCE for compression and also 3–5 times faster for decompression. Furthermore, SRComp remains substantially faster than gzip and bzip2 to compress any dataset tested.

## Discussion

While sequencing reads tend to be longer, the most widely published and adopted next-generation sequencing platform Genome Analyzer IIx is still producing very short reads that are 35, 50 and 75 bases long. Another platform, HiSeq 2000, is designed to generate reads for only three different lengths: 36, 50 and 100 bases. Indeed, short reads are sometimes preferred over long reads (of length 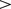
100 bp) because short reads typically have higher data quality than long reads due to their relatively lower sequencing error rates. Therefore, a fast and efficient compression approach is desired to address the pressing issue of storing and transmitting an unprecedented amount of short reads.

In this paper, we presented a new read sequence compression tool called SRComp, which was designed especially for short read sequences. It works with a simple two-stage procedure: it first employs burstsort to sort reads in lexicographical order and then applies Elias omega-based integer coding to the sorted read sequences. In addition, it was implemented in a way that can handle a large volume of data that does not fit in main memory. This is an increasingly important feature as NGS data output continues to grow at an accelerated rate.

A distinguishing advantage of SRComp over other read sequence compression tools is its substantially lower compression time requirement. As shown in our experiments on four real NGS datasets, it can run 4–5 times faster than gzip, 5–8 times faster than SCALCE, and 19–35 times faster than BEETL. We attribute this mainly to the following two reasons. First, burstsort was used to sort read sequences. Burstsort is a cache-conscious algorithm designed specifically for sorting strings. It has been shown to run substantially faster than quicksort on large datasets [14], which is the case for NGS datasets. Second, Elias omega-based integer coding was used to encode reads. To encode a read, only its preceding read (of length 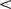
100 bp in our experiments) needs to be accessed and compared to compute a relative integer value. In comparison, dictionary-based coding, which is used in many general-purpose text compressors such as gzip, typically needs to access the last 32K bases prior to a read (i.e., a sliding window) and search for matches. Therefore, it is not hard to see that dictionary-based coding is much more time consuming than Elias omega-based integer coding to encode a read sequence.

While running faster than BEETL and SCALCE by several factors, SRComp can still offer high compression efficiency for short read sequences. In terms of compression bit rates achieved for the four datasets tested above, SRComp performed only 3.80% and 1.49% worse than BEETL and SCALCE, respectively. If compared to the sorting-based method in [10], SRComp actually improved compression efficiency by a significant margin, that is, 21.7% and 23.3% for gzip and bzip2, respectively (see Table 3). We believe that SRComp achieved such high compression efficiency mainly by taking advantage of two characteristics of short reads. First, reads are sorted in lexicographical order, so they can be translated to a monotonic sequence of integers (i.e., 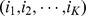 in the Methods section). Second, the NGS data throughput is ultra-high, which implies that a substantially large number of reads have been sampled from a relatively constrained read space when the read length is short. These two characteristics together hence give rise to a sequence of integers (i.e., 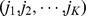 in the Methods section) whose distribution is skewed towards smaller integers (see Figure S1), so that Elias omega coding becomes very efficient for encoding them. In addition, NGS often produces quite a number of identical reads due to enzyme biases, the PCR effect, or the presence of highly repeated sequences in the library (e.g., when highly expressed genes are sequenced at great depth). Compression of these identical reads by Elias omega coding is particularly efficient, requiring only two bits per read.

It should be noticed that the second characteristic mentioned above will gradually disappear as the reads become longer. As a result, SRComp becomes much less efficient with longer reads than other state-of-the-art compression tools such as BEETL and SCALCE, although it remains at high compression speed (see Table 4). A specific reason for this, in comparison to BEETL and SCALCE, is that SRComp basically does not exploit the similarity among reads that are far apart after being lexicographically sorted, e.g., the similarity that occurs between the end of a read sequence and the beginning of another read sequence [10]. This is a limitation of SRComp, limiting its applicability to NGS datasets of long reads especially when high compression efficiency is mainly sought. Nevertheless, SRComp remains more efficient than general-purpose compressors such as gzip and bzip2, which are still the *de-facto* standard for archiving NGS data today.

In conclusion, SRComp is a read sequence compression tool that can run very fast, while for large collections of short read sequences, retaining high compression efficiency achieved by other state-of-the-art read sequence compression tools. It is particularly valuable in certain applications where compression time is of major concern. In future work, we will explore an efficient approach to compressing read quality scores and also implement a multi-threaded version of SRComp.

## Supporting Information

Figure S1 **The frequency distribution of integer numbers used to encode read sequences.**
(PDF)

Table S1 **The run accession numbers of each test dataset.**
(PDF)

## References

[1] Kahn SD (2011) On the future of genomic data. Science 331: 728–729. 21311016 10.1126/science.1197891

[2] Bonfield JK, Mahoney MV (2013) Compression of FASTQ and SAM format sequencing data. PLoS One 8: e59190. 23533605 10.1371/journal.pone.0059190 PMC3606433

[3] Cox AJ, Bauer MJ, Jakobi T, Rosone G (2012) Large-scale compression of genomic sequence databases with the Burrows-Wheeler transform. Bioinformatics 28: 1415–9. 22556365 10.1093/bioinformatics/bts173

[4] Deorowicz S, Grabowski S (2011) Compression of DNA sequence reads in FASTQ format. Bioinformatics 27: 860–2. 21252073 10.1093/bioinformatics/btr014

[5] Fritz MHY, Leinonen R, Cochrane G, Birney E (2011) Efficient storage of high throughput dna sequencing data using reference-based compression. Genome Research 21: 734–740. 21245279 10.1101/gr.114819.110 PMC3083090

[6] Hach F, Numanagic I, Alkan C, Sahinalp SC (2012) SCALCE: boosting sequence compression algorithms using locally consistent encoding. Bioinformatics 28: 3051–7. 23047557 10.1093/bioinformatics/bts593 PMC3509486

[7] Jones DC, Ruzzo WL, Peng X, Katze MG (2012) Compression of next-generation sequencing reads aided by highly efficient de novo assembly. Nucleic Acids Res 40: e171. 22904078 10.1093/nar/gks754 PMC3526293

[8] Kozanitis C, Saunders C, Kruglyak S, Bafna V, Varghese G (2011) Compressing genomic sequence fragments using SlimGene. J Comput Biol 18: 401–13. 21385043 10.1089/cmb.2010.0253 PMC3123913

[9] Tembe W, Lowey J, Suh E (2010) G-SQZ: compact encoding of genomic sequence and quality data. Bioinformatics 26: 2192–4. 20605925 10.1093/bioinformatics/btq346

[10] Wan R, Asai K (2010) Sorting next generation sequencing data improves compression effectiveness. In: IEEE International Conference on Bioinformatics and Biomedicine Workshops. Hong Kong: IEEE Computer Society, 567–572.

[11] Pinho AJ, Pratas D, Garcia SP (2012) GReEn: a tool for efficient compression of genome resequencing data. Nucleic Acids Res 40: e27. 22139935 10.1093/nar/gkr1124 PMC3287168

[12] Popitsch N, von Haeseler A (2013) NGC: lossless and lossy compression of aligned high-throughput sequencing data. Nucleic Acids Res 41: e27. 23066097 10.1093/nar/gks939 PMC3592443

[13] Yanovsky V (2011) ReCoil – an algorithm for compression of extremely large datasets of DNA data. Algorithms Mol Biol 6: 23. 21988957 10.1186/1748-7188-6-23 PMC3219593

[14] Sinha R, Zobel J (2004) Cache-conscious sorting of large sets of strings with dynamic tries. ACM Journal of Experimental Alogirthmics 9: 1–31.

[15] Elias P (1975) Universal codeword sets and representations of integers. IEEE Transactions on Information Theory 21: 194–203.

[16] Cleary JG, Witten IH (1984) Data compression using adaptive coding and partial string matching. IEEE Transactions on Communications 32: 396–402.

