# Supplementary material for: SRComp: Short Read Sequence Compression Using Burstsort and Elias Omega Coding
Source: PLoS One. 2013 Dec 13;8(12):e81414. doi: 10.1371/journal.pone.0081414 (PMC3862494; doi:10.1371/journal.pone.0081414)
Supplement: Figure S1 — The frequency distribution of integer numbers used to encode read sequences. (PDF) [file pone.0081414.s001.pdf]

Figure S1. The frequency distribution of integer numbers used to encode read sequences.

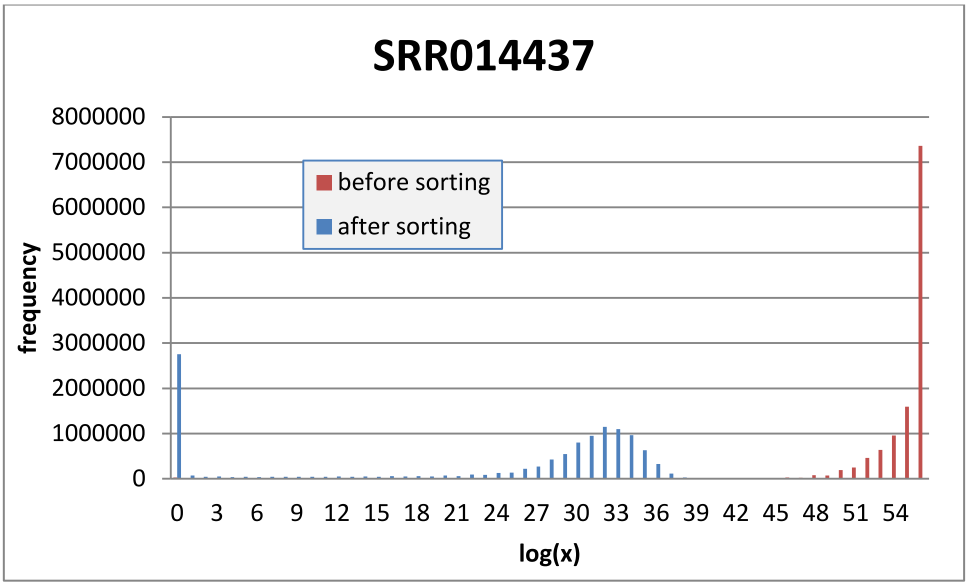

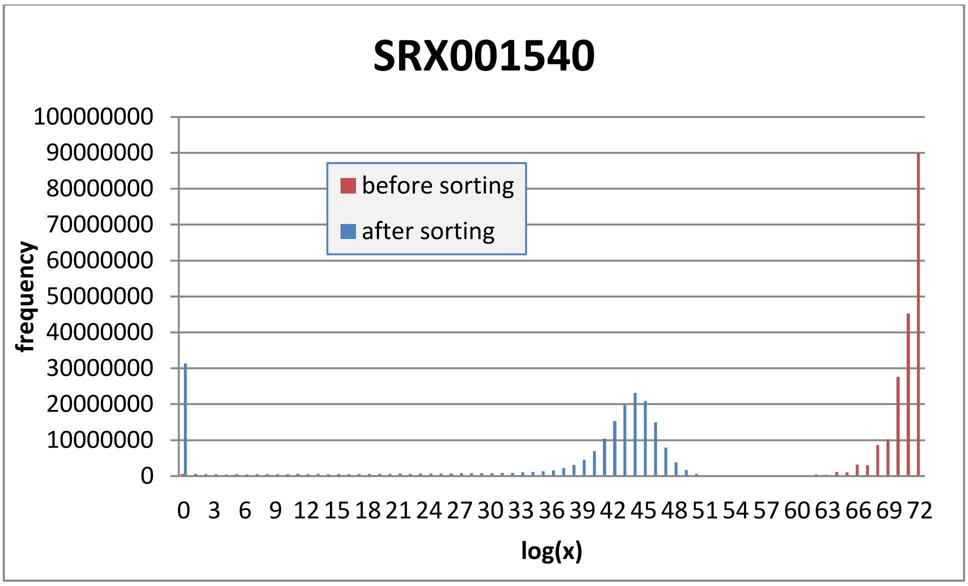

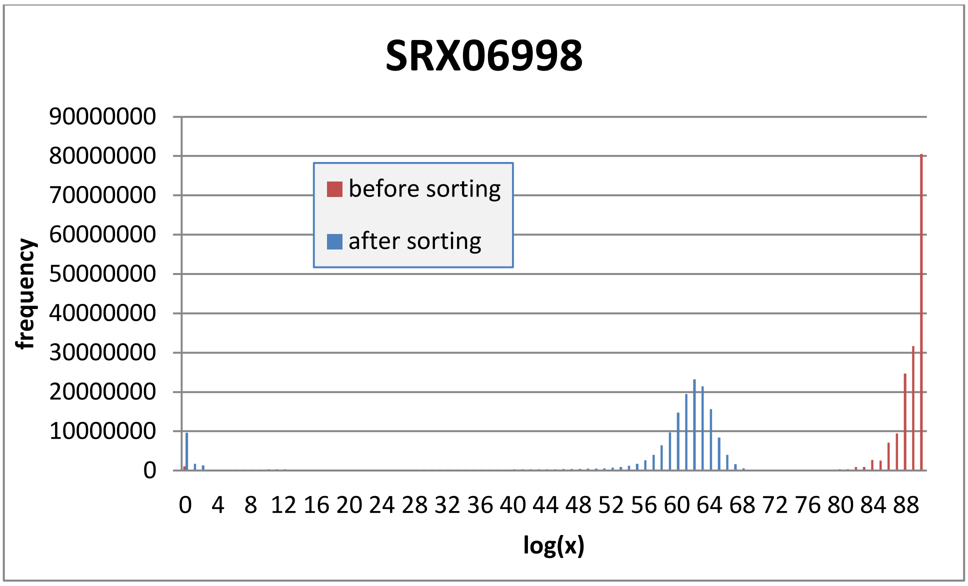

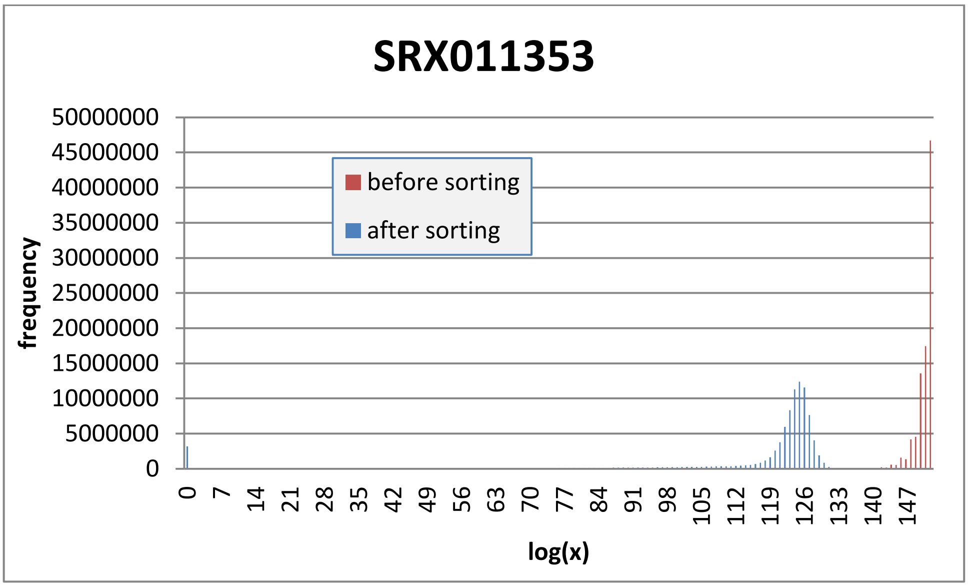

We can observe from the above figures that the main peak of every frequency distribution is shifted to the left towards smaller values after read sequences are sorted in alphabetical order. Here x is used to denote the integers of $(i_1, i_2, \cdots, i_K)$ before sorting but integers of $(j_1, j_2, \cdots, j_K)$ after sorting. For definitions of $(i_1, i_2, \cdots, i_K)$ and $(j_1, j_2, \cdots, j_K)$, please refer to Section “Encoding a collection of sorted reads”.
