# Supplementary material for: SRComp: Short Read Sequence Compression Using Burstsort and Elias Omega Coding
Source: PLoS One. 2013 Dec 13;8(12):e81414. doi: 10.1371/journal.pone.0081414 (PMC3862494; doi:10.1371/journal.pone.0081414)
Supplement: Table S1 — The run accession numbers of each test dataset. (PDF) [file pone.0081414.s002.pdf]

Table S1. The run accession numbers of each test dataset.

SRR014437	SRX001540	SRX006998*	SRX011353	SRX016231
SRR014437	SRR005720	SRR019778	SRR027520	SRR034939
	SRR005721	SRR019779	SRR027540	SRR034940
	SRR005734	SRR019780		SRR034941
	SRR005735	SRR019781		SRR034942
		SRR019782		SRR034943
		SRR019783		SRR034944
		SRR019784		SRR034945
		SRR019785		SRR034946
		SRR019786		SRR034947
		SRR019787		SRR034948
		SRR019788		SRR034949
		SRR019789		SRR034950
		SRR019790		SRR034951
		SRR019791		SRR034952
		SRR019792		SRR034953
		SRR019793		SRR034954
		SRR019794		SRR034955
		SRR019795		SRR034956
		SRR019796		SRR034957
		SRR019797		SRR034958
		SRR019798		SRR034959
		SRR019799		SRR034960
				SRR034961
				SRR034962
				SRR034963
				SRR034964
				SRR034965
				SRR034966
				SRR034967
				SRR034968
				SRR034969
				SRR034970
				SRR034971
				SRR034972
				SRR034973
				SRR034974
				SRR034975

* Only SRR0197??_1.fastq files are included in the tests.
